# Endogenous Symmetric Dimethylarginine (SDMA) and Asymmetrical Dimethylarginine (ADMA) Levels in Healthy Cows and Cows with Subclinical and Clinical Mastitis—A Comparative Study

**DOI:** 10.3390/ani15040527

**Published:** 2025-02-12

**Authors:** Valerio Bronzo, Giulia Sala, Irene Ciabattini, Chiara Orsetti, Giovani Armenia, Valentina Meucci, Lucia De Marchi, Fabrizio Bertelloni, Micaela Sgorbini, Francesca Bonelli

**Affiliations:** 1 Department of Veterinary Medicine and Animal Sciences, University of Milan, Via dell’Università 6, 26900 Lodi, Italy; valerio.bronzo@unimi.it; 2 Department of Veterinary Sciences, University of Pisa, Viale delle Piagge 2, 56124 Pisa, Italy; irene.ciabattini@vet.unipi.it (I.C.); chiara.orsetti@phd.unipi.it (C.O.); giovanni.armenia@phd.unipi.it (G.A.); valentina.meucci@unipi.it (V.M.); lucia.demarchi@unipi.it (L.D.M.); fabrizio.bertelloni@unipi.it (F.B.); micaela.sgorbini@unipi.it (M.S.); francesca.bonelli@unipi.it (F.B.); 3 Centro di Ricerche Agro-Ambientali “E. Avanzi”, University of Pisa, San Piero a Grado, 56122 Pisa, Italy

**Keywords:** dairy cow, udder health, biomarker, ADMA, SDMA

## Abstract

Mastitis is one of the most frequent diseases in dairy farms and occurs in both clinical and subclinical forms, resulting in substantial economic losses. Asymmetrical dimethylarginine (ADMA) and symmetrical dimethylarginine (SDMA) are biomarkers that inhibit nitric oxide synthesis. Elevated ADMA levels are associated with an increased risk of mortality both in human medicine and in dogs and a potential need for intensive care, while SDMA correlates with poor prognoses in humans and the progression of renal disease in horses, though its impact varies depending on renal function. This study examines the plasma levels of ADMA and SDMA in healthy cows (H) and cows with subclinical mastitis (SCM) and clinical mastitis (CM). Cows were classified as having mastitis when CMT > 1 and SCC ≥ 250,000 cells/mL. The SCM group showed no clinical signs or milk alterations, whereas the CM group exhibited udder and/or milk changes. The study included 196 blood samples to determine ADMA and SDMA concentrations, with 96 from healthy cows and 100 from pathological cows (58 SCM and 42 CM). The descriptive statistics were reported as the median because the data were not normally distributed (Shapiro–Wilk test). Data were analyzed using the Kruskal–Wallis test with Bonferroni post hoc correction, and the cut-off and accuracy index were calculated using the gold-standard measurement, the SCC. Statistically significant differences in ADMA levels were observed between healthy cows (0.11 µmol/L) and cows with mastitis (SCM 0.26 µmol/L; CM 0.26 µmol/L), but no differences were found in their SDMA levels. The cut-off for ADMA was >0.164 µmol/L, with a sensitivity of 80.41% and specificity of 77.78%. This study suggests that the blood concentration of ADMA is statistically higher in cows with subclinical and clinical mastitis and could be further explored as a potential biomarker for diagnosing these diseases.

## Simple Summary

Mastitis, a common infection of the udder, is a challenge in dairy cows due to its different forms, including subclinical and clinical mastitis, as well as the economic losses and the use of antimicrobials associated with it. For this reason, interest in biomarkers for mastitis has increased in recent years. Asymmetric dimethylarginine and symmetric dimethylarginine, which have shown promising results as biomarkers for various diseases, are explored in this study for detecting mastitis in dairy cows. The study involved 196 Holstein cows, including healthy cows and those with subclinical and clinical mastitis. The results underlined that ADMA levels were higher in cows with mastitis, and it could be used as a reliable marker to differentiate healthy cows from those affected by the disease. However, SDMA did not show significant differences between the groups, suggesting it may not be as useful for diagnosing mastitis. These findings suggest that ADMA could be a biomarker for the detection of subclinical and clinical mastitis ultimately benefiting both cow health and the dairy industry.

## 1. Introduction

Mastitis is an inflammatory condition of the mammary gland caused by various pathogens, including bacteria, viruses, fungi, and algae [1]. In some cases, it can also result from an imbalance in antioxidant defenses, leading to inflammatory oxidative stress [2,3]. This condition can cause tissue damage, adversely affect animal welfare, and lead to increased use of antimicrobial drugs, which all contribute to significant economic losses in the dairy industry [1]. Mastitis manifests in two main forms: clinical mastitis, which is characterized by visible changes in both the milk and the udder, and subclinical mastitis, which does not show obvious changes in the milk but is linked to reduced milk production, altered milk composition, and elevated somatic cell count (SCC) [1,4]. Subclinical mastitis is notably more prevalent, occurring 15 to 40 times more frequently than clinical mastitis [5].

The challenge of diagnosing subclinical mastitis lies in the absence of overt clinical signs, making it difficult to detect without laboratory-based methods [6]. The most common diagnostic approach is measuring the SCC in milk, with varying thresholds that differ in sensitivity and specificity [6,7]. Other methods, though less accurate, include the analysis of milk lactose concentration [8], levels of enzymes such as lactate dehydrogenase and N-acetyl-β-D-glucosaminidase [9], acute-phase proteins like haptoglobin and amyloid A [10], and milk electrical conductivity [11,12]. Despite their widespread use, these traditional on-farm diagnostic methods often lack the sensitivity needed to reliably identify subclinical mastitis [6], which has sparked growing interest in evaluating metabolites and biomarkers associated with the inflammatory processes that occur in mastitis [5,13,14,15]. These biomarkers could potentially offer more accurate and reliable diagnostic tools, leading to improved management strategies and outcomes in dairy farming. For this reason, in recent years, new biomarkers that have shown promising results in human medicine and in other animal species have been investigated in cattle, specifically for the diagnosis and management of mastitis [13].

Asymmetric dimethylarginine (ADMA) is an endogenous inhibitor of nitric oxide (NO) synthesis that acts non-specifically [16]; it uncouples NO synthase (NOS) isoenzymes to produce a superoxide that contributes to increases in oxidative stress [17]. ADMA inhibits NO synthesis by competing for the conversion of the catalytic substrate [18]. NO derives from the metabolism of L-arginine by NO synthase (NOS) [19]; arginine influences the immune system, promoting the polarization of macrophages, which secrete proinflammatory cytokines and phagocytize bacterial pathogens [5].

The study by O’Dwyer et al. [20] shows that the ADMA-mediated inhibition of inducible NOS (iNOS) in patients with sepsis may interfere with macrophage bactericidal properties. Symmetric dimethylarginine (SDMA) decreases NO synthesis at the endothelial level by inducing intracellular arginine deficiency, since SDMA competes with the cationic amino acid transporter in the endothelial cell membrane [21]. SDMA also shows a proinflammatory effect by enhancing the differentiation and adhesion capacity of leukocytes to the endothelium and it induces reactive oxygen species (ROS) in monocytes [17]. Variations in NO levels are correlated with both circulatory failure and infection control [22,23]. SDMA does not directly inhibit the enzyme NOS but interferes with NO synthesis by competing with arginine, inhibiting its cellular uptake [18,24].

ADMA and SDMA both originate from the intranuclear methylation of L-arginine residues and are released into the cytoplasm following proteolysis, which suggests that ADMA and SDMA are constantly produced during normal protein turnover [21]. The production of ADMA is balanced by its metabolism by dimethylarginine dimethylaminohydrolase (DDAH), so an impairment of DDAH function, executed by oxidative stress, is a central mechanism of increases in ADMA levels and its accumulation [25].

In the study by Zwierzchowski et al. [14], the urinary concentration of SDMA rises in cows with subclinical mastitis, showing its potential as a sensible marker for mastitis.

Given the limited literature on the use of ADMA and SDMA as biomarkers in mastitis, this study aims to evaluate the plasmatic concentrations of these biomarkers in healthy cows, as well as in cows affected by clinical and subclinical mastitis. It is hypothesized that the plasmatic concentrations of ADMA and SDMA will be higher in cows with mastitis compared to healthy animals, and that these differences could potentially aid in the diagnosis of mastitis.

## 2. Materials and Methods

### 2.1. Study Design

Lactating Italian Holstein cows belonging to the herd of Centro di Ricerche Agro-ambientali “E. Avanzi” of the University of Pisa were enrolled for a comparative study on the mammary gland health between January 2021 and December 2023. The approval for the present study was obtained from the Institutional Animal Care and Use Committee, University of Pisa (Protocol N: 18/2023 of 19 April 2023).

The sample size was determined using an ANOVA test analysis to identify potential differences in ADMA and SDMA concentrations among healthy, SCM, and CM animals (G-power v. 3.1, Heinrich-Heine-Universität, Düsseldorf, Germany). An effect size of 0.3, a type I error (α) of 5%, a confidence interval of 95%, and a test power of 95% were applied, resulting in a minimum sample size of 178 cows. Due to the lack of studies on ADMA and SDMA in bovine species, the minimum number of animals was increased by 10%, bringing the final number included in the study to 196 animals.

### 2.2. Animals and Management

Animals underwent the same feeding and management practices. Cows were kept in a free-stall barn with permanent straw bedding which was completely replaced every 3-4 days, and an addition of clean straw was performed daily. Their access to fresh water was free and feed was supplied twice a day as a total mixed ration. Milking procedures were performed two times a day, using a Herringbone milking parlor. Udder health monitoring was performed through regular veterinary checks conducted once a week, consisting of somatic cell count (SCC) and clinical evaluation. The same veterinary service was also called upon by the milkers for the evaluation of the animals each time there was a macroscopic alteration in the milk.

### 2.3. Inclusion Criteria

Each week during the udder health monitoring program, the cows were evaluated and recorded as healthy or pathologic based on a clinical examination plus udder and milk evaluations. Examinations were performed by the veterinarian before the milking procedures were carried out (afternoon), while udders and teats were inspected and palpated for any signs of inflammation once the cow entered the milking parlor [26,27]. Before milking, California Mastitis Test (CMT) and somatic cell count (SCC) (DCC DeLaval^®^, DeLaval s.p.a., Milan, Italy) evaluations were carried out at the quarter level on the farm. Animals were classified as healthy (H) if they showed no systemic or local signs of inflammation, CMT < 1 [28], and an SCC < 100.000 cell/mL for primiparous cows or an SCC < 200.000 cell/mL for pluriparous cows [7,29]. Animals were classified as affected by subclinical mastitis (SCM) if they showed at least one quarter of the udder with no systemic or local sign of inflammation, CMT > 1 [28], and an SCC > 100.000 cell/mL for primiparous cows or an SCC > 200.000 cell/mL for pluriparous cows [7,29]. For clinical mastitis, mild, moderate, and severe cases were included. Specifically, clinical mastitis (CM) was diagnosed in cows that had at least one quarter of the udder producing altered milk (e.g., milk clots or serous secretions), with or without visible udder changes such as swelling, hardness, warmth, or pain and systemic signs [30]. Cows showing signs of other comorbidities, such as lameness, were excluded.

### 2.4. Sampling

Sampling procedures were performed in cows included in all the three groups. Milk samples were collected at the quarter level following the National Mastitis Council (NMC) guidelines [31]. Before sampling, a thorough cleaning of the teat was conducted using a pre-dipping foam containing lactic acid (Biofoam Plus, DeLaval Inc., Tumba, Sweden). The teat was then dried, and the apex was disinfected using alcohol [32]. Two to three streams of foremilk were discarded [33], and approximately 10 mL of milk was aseptically collected in sterile vials. Samples were stored at 4 °C until the bacteriological analyses were carried out. For the bacteriological analysis, the milk samples were processed in accordance with NMC guidelines [34]. Specifically, ten microliters of each milk sample were spread on 5% defibrinated sheep blood agar plates (Microbiol, Cagliari, Italy) and incubated aerobically at 37 °C for 24–48 h. The plates were subsequently evaluated and categorized as positive, contaminated, or negative based on NMC criteria [31]; in particular, samples exhibiting the growth of three or more different colonies were considered contaminated [34]. From positive plates, colonies were purified on 5% defibrinated sheep blood agar plates and the obtained isolates were identified using MALDI-TOF MS [35], with their spectra analyzed using the MALDI Biotyper (MBT) Compass^®^ Library Revision H (2022) (Bruker Daltonik GmbH, Bremen, Germany). A custom MALDI-TOF MS library was employed, incorporating two *Staphylococcus rostri* strains previously identified via *rpoB* gene sequencing [36]. Samples with three or more pathogens were considered contaminated [31].

Blood was drawn from the coccygeal vein into a lithium heparin test tube (FL Medical, Torreglia, Italy) after the milking session. Samplings were immediately centrifuged at 3000 rpm for 10 min (Legend RT, Sorvall; ThermoFisher Scientific Inc., Waltham, MA, USA) and the obtained plasma was divided into 4 aliquots which were frozen at −80 °C until analysis.

### 2.5. ADMA and SDMA Concentration Determination

At the Veterinarian Pharmacology and Toxicology Laboratory of the Department of Veterinary Science, University of Pisa, the concentration of ADMA and SDMA was evaluated using high-performance liquid chromatography (HPLC) with fluorescence detection, following the Teerlink [37] method. A standard solution was prepared with stock solutions containing 1 mM of ADMA, and SDMA in 10 mmol HCl. Aliquots were stored at −20 °C and thawed prior to analysis. The derivatization agent was prepared with 10 mg orthophthalaldehyde (OPA) dissolved in 0.2 mL methanol, 1.8 mL potassium borate buffer (pH 9.5), and 10 µL of 3-mercaptopropionic acid, which was diluted fivefold shortly before derivatization to prepare the working solution. Samples and standards underwent solid-phase extraction using a vacuum system, where 0.2 mL of each sample or standard was combined with 0.8 mL of PBS. Columns were used without preconditioning, and washing steps involved 1.0 mL of 100 mmol HCl and 1.0 mL of methanol. Analytes were eluted with 1.0 mL concentrated ammonia–water–methanol (10:40:50) (*v/v*), and the solvent was removed by nitrogen evaporation. Subsequently, 0.1 mL of OPA reagent was added, and samples were transferred to autosampler vials. Chromatographic separation was carried out on a Novapack (C18, 5 micron, 4.6 mm × 150 mm, Waters, Milford, MA, USA) column with a mobile phase consisting of potassium phosphate buffer (pH 6.5) with acetonitrile and water as solvents. Fluorescence was detected at excitation and emission wavelengths of 340 and 455 nm. The intra- and inter-assay coefficients of variation for SDMA and ADMA were less than 11% and 15%, respectively, with lower limits of quantification set at 0.05 µmol/L for ADMA and 0.02 µmol/L for SDMA, using a sample volume of 0.2 mL. Analyses were conducted in batches of 30 samples.

### 2.6. Statistical Analysis

The concentrations of biomarkers obtained were organized into a spreadsheet (Microsoft Excel, Office Suite) and imported into SPSS 29 (IBM, Armonk, NY, USA) for statistical analysis. Quantitative variables were reported as the median, 25th, and 75th percentiles since the data were not normally distributed (Shapiro–Wilk test). Qualitative variables were reported as frequency and percentage.

To evaluate whether one or both biomarkers could differentiate between healthy animals, those with subclinical mastitis, or those with clinical mastitis, the Kruskal–Wallis test was used, and post hoc comparisons were performed with Bonferroni correction. To assess if the parity, days in milk (DIM), and body condition score (BCS) were different in the healthy cows or the cows with subclinical, or clinical mastitis, all cows were compared using the Kruskal–Wallis test, followed by Bonferroni’s post hoc test for DIM, while the chi square test was used for parity and BCS. Significance was set at a *p*-value < 0.05.

For biomarkers that were statistically different between healthy animals and those with mastitis, a cut-off was calculated using the ROC (Receiver Operating Characteristic) curve divided between subclinical and clinical mastitis. The optimal cut-off was chosen using the Youden index, in which the sensitivity and specificity are maximized, with equal weight given to false positive and false negative results [38]. The calculated cut-off values were then used to determine sensitivity (Se), specificity (Sp), positive predictive value (PPV), negative predictive value (NPV), and test accuracy. Additionally, the area under the curve (AUC) and its 95% confidence interval (CI) were calculated and used as indicators of biomarker accuracy. The interpretation of the AUC is based on 1.00 for a perfect test, 0.99–0.90 for an excellent test, 0.89–0.80 for a good test, 0.79–0.70 for a fair test, 0.69–0.51 for a poor test, and 0.50 for a failed test [39].

## 3. Results

For the evaluation of ADMA and SDMA, a total of 196 samples were analyzed, which were distributed as follows: 96 from healthy cows and 100 from cows with mastitis (58 animals with subclinical mastitis; 42 animals with clinical mastitis). The population included in the study had a DIM of 94 (55–208) and BCS of 3.0 (3.0–3.25). In the study population, 66 (33.7%) cows were primiparous, 71 (36.2%) secundiparous, and 59 (30.1%) cows were on their third or more lactation.

All the H animals tested negative for bacteriological analysis, while 23% (23/100) of the animals tested positive in mastitis groups, 15.5% (9/58) of the SCM cows and 33.3% (14/42) of the CM animals; in each mastitis group there was a case in two different colonies were isolated from a single milk sample. For the SCM group, the isolated bacteria were non-aureus staphylococci and mammaliicocci (NASM) (5/58, 8.6%), *Streptococcus dysgalactiae* (2/58, 3.4%), *Enterococcus faecium*, *Enterococcus* spp., and *Aerococcus viridans* (each representing 1/58, or 1.7%). For the CM group, the isolated bacteria were NASM (6/42, 14.3%), *Escherichia coli* (3/42, 7.1%), *Aerococcus viridans*, *Enterobacter cloacae*, *Serratia odorifera*, *Staphylococcus aureus*, and *Streptococcus uberis* (each 1/42, 2.4%). Of the 42 cases of clinical mastitis, 32 (76.2%) were classified as mild, 8 (19.0%) as moderate, and only 1 (4.8%) as severe.

The results of plasmatic ADMA and SDMA concentrations in H, SCM and CM groups are presented in Table 1.

Statistically significant differences were observed between the plasmatic ADMA concentrations in the H and SCM groups (*p* < 0.001), as well as between the H and CM groups (*p* < 0.001); no significant differences were found between the SCM and CM groups. No statistically significant differences were observed in the SDMA concentrations among the various study groups.

The non-parametric test between groups revealed no statistically significant differences for days in milk (DIM), parity, or body condition score (BCS). For DIM, the H group had a median of 180 days (55–250), the SCM group had a higher median of 200 days (60–296), and the CM group had a median of 160 days (76.50–257.75), with no significant differences among the groups (*p* = 0.096). Regarding parity, the H group consisted of 44.8% (43/96) primiparous cows, 45.8% (44/96) secundiparous cows, and 9.4% (9/96) cows in their third or higher lactation. Similarly, the SCM group had 39.7% (23/58) primiparous, 47.3% (27/58) secundiparous, and 13.0% (8/58) on their third or more lactation, while the CM group had 45.4% (19/42) primiparous, 41.2% (17/42) secundiparous, and 13.4% (6/42) on their third or more lactation, with no significant differences observed across the groups (*p* = 0.106). For BCS, the H group had 39.6% (38/96) of cows with a score of 3.00, 25.0% (24/96) with a score of 3.25, and 10.4% (10/96) with a score of 2.75. In the SCM group, 48.3% (28/58) had a BCS of 3.00, 15.5% (9/58) had a score of 3.25, and 13.8% (8/58) had a score of 2.75. Similarly, the CM group had 38.1% (16/42) of cows with a BCS of 3.00, 23.8% (10/42) with a score of 3.25, and 16.7% (7/42) with a score of 2.75. No significant differences were found between the groups for BCS (*p* = 0.870).

**Figure 1:**
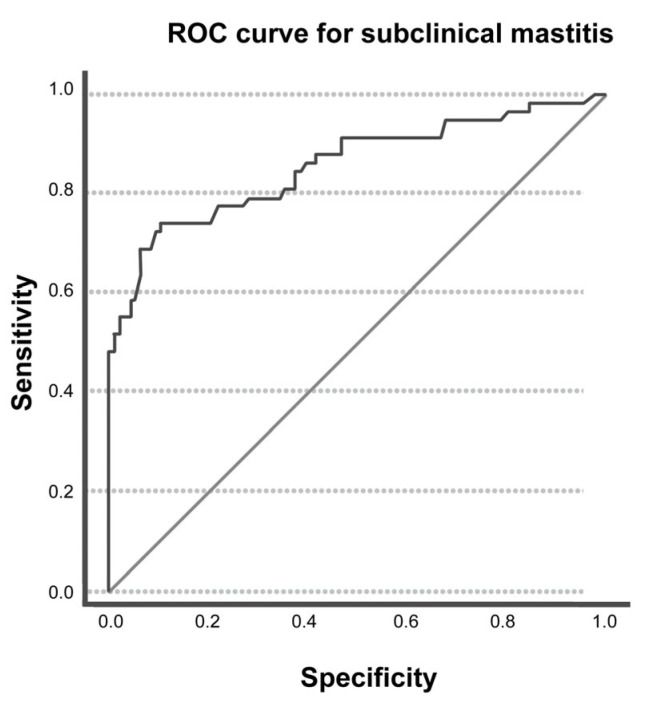
Receiver operating characteristic (ROC) and area under the curve (AUC) for the optimal threshold of ADMA to distinguish between the presence and absence of subclinical mastitis.

For the subclinical mastitis, the cut-off obtained through the ROC analysis was >0.164 µmol/L with an AUC of 0.857 (0.790; 0.924) (Figure 1). The sensitivity and specificity of ADMA were found to be 72.41% (CI: 59.10% to 83.34%) and 89.58% (CI: 81.68% to 94.89%), respectively. The PPV and the NPV for ADMA were 80.77% (CI: 69.58% to 88.52%) and 84.31% (CI: 77.89% to 89.13%), respectively.

**Figure 2:**
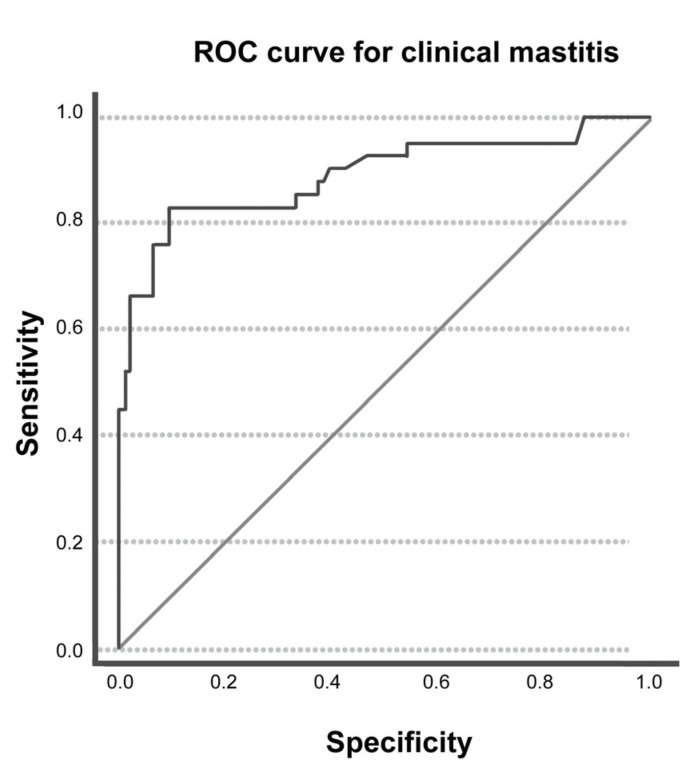
Receiver operating characteristic (ROC) and area under the curve (AUC) for the optimal threshold of ADMA to distinguish between the presence and absence of clinical mastitis.

For clinical mastitis, the cut-off obtained through the ROC analysis was >0.169 µmol/L with an AUC of 0.892 (0.824; 0.961) (Figure 2). The sensitivity and specificity of ADMA were found to be 83.33% (CI 68.64%; 93.03%) and 89.58% (CI 81.68%; 94.89%). The PPV and NPV for ADMA were found to be 77.78% (CI 65.62%; 86.47%) and 92.47% (CI 86.16%; 96.04%).

## 4. Discussion

In our study, we found that plasmatic ADMA can differentiate healthy cows from those affected by mastitis, both subclinical and clinical, while no discriminatory effectiveness has been attributed to SDMA. There are few studies evaluating ADMA and SDMA in cattle, with most analyzing these biomarkers in both blood and urine, including mastitis but often focusing on other diseases and studying them before rather than during the disease [5,14,15,40,41,42,43]. Furthermore, the results obtained are discordant, and the behavior of ADMA and SDMA is not consistent across studies [5,14,15,40,41,42,43].

In our study, the ADMA concentration increased in the SCM and CM cows compared the to H cows. The increase in ADMA, both in blood and urine, has been highlighted by other studies 8 and 4 weeks before calving in cows that later developed subclinical mastitis [5,43]. The only study reporting a variation in ADMA during the subclinical mastitis episode is the study by Zhang et al. [5]. In this study, the serum ADMA levels were higher 8 and 4 weeks before parturition in cows that later developed subclinical mastitis, but lower in these cows at the time of diagnosis compared to the control group. No explanation was provided regarding the timing of sampling in relation to the diagnosis in their study, which is addressed in ours. In fact, all cows in the SCM and CM groups in our study were sampled on the same day as their diagnosis. A possible explanation for these contrasting results could be that blood ADMA levels rise in the early stages of the disease and decrease as it resolves, since ADMA inhibits NOS only at high concentrations [44], and ADMA levels are rapidly reduced by the enzyme DDAH [37,45]. The possibility that prolonged elevated blood ADMA levels may be harmful has been highlighted in many studies [46,47]. In fact, prolonged elevated levels of ADMA have been correlated with the prognosis of a specific disease. Serum concentrations of ADMA have been primarily examined in human patients with sepsis, showing that ADMA can be used as a prognostic indicator. Some studies highlighted that the levels of ADMA in plasma differentiate healthy individuals from pathological ones and an elevated concentration of ADMA identified individuals at risk of mortality and potentially in need of intensive care [46,47]. ADMA is the direct inhibitor of NO, which is a free radical gas and it plays a crucial role in the body’s defense as a cytotoxic agent [48]. It has immediate antimicrobial effects, including the breakdown of bacterial target structures and the inhibition of bacterial metabolism [49,50]. NO derives from the metabolism of L-arginine by NO synthase (NOS) [19], arginine influences the immune system, promoting the polarization of macrophages, which secrete proinflammatory cytokines and phagocytize bacterial pathogens [5]. The inducible form of NO synthase is primarily expressed in immune tissue and is capable of producing high amounts of NO during inflammation [51]. This may explain the adverse effects of an elevated level of circulating ADMA on inflammatory response.

The differences in the study design and size of the population between our study and that of Zhang et al. [5] may explain the different results found. Another possible explanation is that we included both clinical and subclinical mastitis, whereas Zhang et al. may have focused on subclinical mastitis only, using a single cut-off of 200,000 cells/L to categorize cows with or without disease. Other explanations may be the different blood matrices used, or the type of analysis used; indeed, Zhang et al. employed metabolomics, which has a different sensitivity to HPLC [52,53].

Concerning SDMA, in our study, the plasmatic concentration of SDMA was the same in the healthy and pathological cows. Zhang and colleagues [5] found that SDMA tends to increase in the week of subclinical mastitis diagnosis, which occurred after calving, compared to 8 and 4 weeks prior to parturition, but the values are not statistically significant enough to assert that this BIO increases with the onset of the pathological event, nor to attribute its potential use as a discriminating marker. This result is consistent with what was observed in our study.

Limitations of our study may include the unintentional inclusion of animals with other subclinical diseases in the control group. However, the chance of including unhealthy animals in the control group is considered very low, since the herd from which the animals came was subjected to weekly screening for various diseases and/or clinical/subclinical conditions by the veterinary service of the institution. Another limitation is that ADMA and SDMA were measured only in plasma and not in milk or urine, which could represent biological matrices that are easier to collect, even by untrained personnel.

The diagnostic accuracy results in our study should be interpreted with caution due to potential sources of bias associated with the study design [54]. Case–control approaches have been shown to overestimate diagnostic accuracy compared to cross-sectional designs, often leading to artificially inflated sensitivity and specificity estimates [55]. This overestimation is largely due to spectrum effects, as the restricted selection of cases and controls does not fully reflect the clinical variability observed in routine practice [56]. In our study, the inclusion of cases with more advanced stages of subclinical mastitis may have led to an overestimation of sensitivity, while the selection of controls without concurrent inflammatory conditions may have resulted in an overestimation of specificity due to a limited challenge bias [57]. To mitigate these concerns, future studies should prioritize cross-sectional designs that better represent real-world diagnostic scenarios and minimize spectrum bias. Additionally, incorporating alternative biological matrices such as milk or urine could enhance diagnostic accuracy and applicability by reducing the risk of selection bias and improving test performance in diverse clinical settings.

## 5. Conclusions

In conclusion, the present study found that ADMA levels differ between pathological and healthy cows under the experimental conditions adopted. In particular, the concentration of plasmatic ADMA was higher in animals with mastitis, both in subclinical and clinical cases. No differences were seen between cows affected by subclinical mastitis and those affected by clinical mastitis. This biomarker could be used for the diagnosis of mastitis, but evidence linking BIOs to the severity of the inflammatory process is lacking. We did not find any variation in the SDMA levels in our population, suggesting the absence of diagnostic effectiveness for this biomarker.

However, our findings are limited to the specific experimental conditions used in this study. Further cross-sectional studies on a larger population are needed to confirm these results and to explore the relationship between these BIOs and the pathogens involved in mastitis.

## Figures and Tables

**Table 1:** Median (25th percentile and 75th percentile) of asymmetrical dimethylarginine (ADMA) and symmetrical dimethylarginine (SDMA) plasmatic concentrations in healthy, subclinical mastitis, and clinical mastitis cow groups.

	Healthy (*n* = 96)	Subclinical Mastitis (*n* = 58)	Clinical Mastitis (*n* = 42)
ADMA (µmol/L)	0.11 (0.09–0.15) ^a^	0.26 (0.15–0.37) ^b^	0.26 (0.18–0.36) ^b^
SDMA (µmol/L)	0.11 (0.08–0.14) ^a^	0.10 (0.07–0.14) ^a^	0.08 (0.06–0.15) ^a^

Groups with different superscript letters (a or b) were statistically different from each other (*p* < 0.05). In contrast, those with the same superscript letters were not statistically different from each other (*p* > 0.05).

## Data Availability

The datasets generated and analyzed during the current study are available from the corresponding author upon reasonable request.
